# Reference Values for Interleukin-6 and Interleukin-8 in Cord Blood of Healthy Term Neonates and Their Association with Stress-Related Perinatal Factors

**DOI:** 10.1371/journal.pone.0114109

**Published:** 2014-12-08

**Authors:** Daan Barug, Susan Goorden, Martien Herruer, Moira Müller, Richard Brohet, Peter de Winter

**Affiliations:** 1 Department of Pediatrics, Spaarne Hospital, Hoofddorp, The Netherlands; 2 Atal-Medial Medical Diagnostic Centers, Hoofddorp, The Netherlands; 3 Department of Obstetrics and Gynaecology, Spaarne Hospital, Hoofddorp, The Netherlands; 4 Research Center Linnaeus Institute, Spaarne Hospital, Hoofddorp, The Netherlands; University of Florida, United States of America

## Abstract

**Background:**

Automated interleukin assays are promising diagnostic aids for early-onset neonatal sepsis, however, reference values for healthy term neonates are incompletely known. The goal of this study is to determine reference values for interleukin-6 (IL-6) and interleukin-8 (IL-8) in cord blood of healthy term neonates.

**Methods and Findings:**

Women were recruited from April 2012 to August 2012. IL-6 and IL-8 levels were measured using an automated immunometric assay (Immulite) in cord blood of 93 healthy term newborns, 60 of them were born via vaginal delivery and 33 by elective caesarean section (ECS). A mean value for IL-8 of 8.1±3.0 pg/mL was found in cord blood of healthy term neonates, which apply to both vaginal delivery and ECS. Regarding IL-6, two values apply. For vaginal delivery, a median value of 3.3 pg/mL (range, <2 to 9.53 pg/mL) was found, while for ECS, a median value of <2 pg/mL (range, <2 to 48 pg/mL) applies.

**Conclusions:**

We propose a reference value of <14.1 pg/mL for IL-8 (mean + 2SD), applying to vaginally delivered and ECS-delivered healthy term newborns. From a clinical point of view, we also propose one reference value for IL-6 to be applied to vaginally delivered and ECS-delivered healthy term newborns, which is <10.2 pg/mL (97.5th percentile total group). These values have to be validated in larger cohorts of neonates, inclusive of those with and without early-onset neonatal sepsis.

## Introduction

Neonates are highly susceptible to infections. Early-onset neonatal bacterial infection occurs within 72 h of birth and is usually due to infection of maternal origin [1]. Prompt and accurate diagnosis and treatment of newborns experiencing an early-onset neonatal sepsis (EONS) is crucial. Although such an infection may appear harmless at the start, it can induce a septic shock very rapidly and is associated with serious morbidity and even mortality [2]. Suspicion of EONS may be based on various anamnestic or clinical risk factors including maternal fever, fever in the newborn and problems in breathing or circulation in the newborn. Clinical signs of infection in the newborn are unspecific and often difficult to distinguish from the physiologic changes that occur during transition to extra uterine life [3]. Moreover, other non- infectious processes, such as transient tachypnea of the newborn, environmentally induced fluctuation of body temperature, and apnea of prematurity are also associated with similar manifestations [4].

The diagnostic procedure for the detection of EONS involves execution of a blood or spinal fluid culture. Measurement of C-reactive protein (CRP) in neonatal serum is an approach to identifying newborns at high risk of EONS while awaiting culture results. However, measurement of CRP has some restrictions and is therefore not ideal in the acute setting of EONS. The timing of obtaining samples is critical to achieve optimal sensitivity [5]. In addition, the specificity of CRP is low for EONS, due to its association with other conditions, for example maternal fever during labour, fetal distress and vacuum extraction [6]. Blood cultures fail to detect bacteraemia in an appreciable number of cases, due to the transient and intermittent nature of bacteremia in the neonate, especially during the early stages of infection. Moreover, they may fail because insufficient amounts of blood have been collected for culture, or processing of specimens is suboptimal [7]. Finally, incubation of bacteria takes 24–48 h, hampering its use in the acute phase.

As EONS can be fulminant in character, antibiotic therapy is usually administered even before diagnostic results are available. As a result, it has been estimated that for every culture proven infection, 11 to 28 non-infected newborn infants are treated unnecessarily with antibiotics [8]. Hence, there is much interest in alternative markers with a more optimal diagnostic profile that are able to rapidly detect an infection. Such markers can help reducing the use of antibiotics and avoid hospital admissions. Among others, proinflammatory cytokines such as interleukin-6 (IL-6) and -8 (IL-8) have been suggested as novel diagnostic markers, as they have shown an increase within a few hours after onset of infection in neonates [6], [9]–[11]. Both interleukins are early pro-inflammatory cytokines predominantly produced by monocytes, macrophages, and endothelial cells. IL-6 promotes CRP synthesis by liver cells, while IL-8 mainly acts as a chemo-attractant for granulocytes [12]. Importantly, Kurt et al showed that IL-6 and IL-8 levels were significantly raised in serum of newborns with clinical signs and a culture-proven sepsis compared to healthy, non-infected newborns [2]. In addition, Franz et al illustrated a reduction in postnatal antibiotic therapy when IL-8 measurement is added to CRP in the diagnostic strategy [13]. In a significant number of neonates with EONS, infection starts in utero resulting in elevated levels of IL-6 and IL-8 in cord blood [3], [10], [14]–[17]. Collection of cord blood is non-invasive and convenient. Therefore, cord blood may offer an alternative to neonatal serum for early diagnosis of EONS.

Reference values are an important issue in judging diagnostic markers. In cord blood, reference values for IL-6 and IL-8 are incompletely known. In most studies, control groups were small and consisted of neonates with risk factors of EONS instead of healthy neonates [3], [14], [16]. Moreover, in some of these studies manual assays, that are not suitable for implementation in the acute diagnostic work-up of EONS, were used [15], [17]. Therefore, the goal of this study was to determine IL-6 and IL-8 reference values in cord blood of healthy term neonates using an automated assay. Cord blood is the earliest hematologic sample taken from the neonate, which could guide the clinicians to carry out effective therapeutic strategy as soon as possible [10]. We aimed to establish reference values for newborns that would apply to both vaginal delivery and elective caesarean section (ECS). In addition, we measured interleukin levels in a subgroup of newborns born via vaginal delivery combined with epidural analgesia or vacuum extraction to study potential influences of these perinatal stress factors on interleukin levels. Notably, women receiving epidural analgesia are at risk of developing fever that is not associated with a neonatal infection, but hard to distinguish from it. This fever is associated with elevated IL-6 [18].

## Materials and Methods

### Study design and participants

From April 2012 to August 2012, healthy term neonates born via vaginal delivery or by ECS at the Spaarne Hospital, Hoofddorp, The Netherlands, were enrolled into NESCIO Project, a study on reference values of neonatal interleukins. According to the Dutch regulation for research with human subjects, neither medical nor ethical approval was required to conduct the study since the present study was purely explanatory and did not include additional medical actions or interventions. Therefore, approval was received from the institutional review board of the Spaarne Hospital (Advice Committee Practicability of the science agency Linnaeusinstitute (ACLU) and all mothers provided written informed consent.

Exclusion criteria were gestational age <37 weeks, clinical signs of infection within 72 h after birth, syndromal abnormalities, fetal tachycardia (≥160/min), suspicion of ABO hemolytic disease of the newborn or rhesus isoimmunization, prolonged rupture of membranes (>24 h), maternal fever (>37.8°C) or maternal tachycardia (≥100/min) during labour, meconium stained amniotic fluid, maternal leukocytosis ≥15×10^6^/L, cervical cerclage or conization with rupture of membranes, perinatal infections (TORCHES) or maternal colonisation with Group B *Streptococcus*. In addition, neonates with a combination of stress-related perinatal factors such as epidural analgesia and vacuum extraction, were excluded.

Characteristics of mother and child were collected. Maternal characteristics included were age, number and outcome of pregnancies, gestational age at delivery, temperature during labour, use of antibiotics during pregnancy or labour, suspicion of rhesus disease, use of corticosteroids, time between collection and freezing samples, use of analgesia (pethidin or regional anesthesia during ECS) and medical indication. Characteristics of the child included sex, birth weight, Apgar score, and temperature as well as admission to neonatology, if needed. In addition, 3 days after birth the child's health status was checked with emphasis on clinical signs of infection.

### Sample collection

Immediately after birth, cord blood (with a maximum of 7 mL) was collected in regular serum tubes (Vacutainer Tubes; BD, Franklin Lakes, New Jersey, USA). Blood was allowed to coagulate for at least 30 min. After centrifugation, the obtained serum was aliquotted in cryotubes (NunC; Thermo Scientific, Waltham, Massachusetts, USA) with a volume of 0.5 mL per tube and frozen at −80° C. Measurements were done after a maximum delay of 3 months. Aliquots were freshly thawed shortly prior to analysis.

### Blood sample analysis

#### Interleukin-6 and -8

IL-6 and IL-8 (350 µL serum) were measured by an automated solid-phase chemiluminescent immunometric assay (Immulite 1000; Siemens, Munich, Germany) that delivers results in 60 and 30 min for IL-6 and IL-8, respectively. For this study, the assay was validated in our laboratory. In the validation phase, control serum-based material provided by Siemens and patient serum samples were used. A CLSI EP10 protocol was used to perform the initial validation in which 3 samples containing a high, medium and low concentration of the metabolite were analyzed in the fixed sequence of mid, high, low, med, med, low, low, high, high, med for 5

days, yielding a total mean CV of 4% for IL-8 and a total mean CV of 6.5% for IL-6. Similar CV scores were obtained using patient samples. After the validation, study samples were analyzed in batches, containing approximately 30 samples and during every run control serum-based material was taken along as a means of quality control. The limit of detection (LOD) of both assays is 2 pg/mL. Values below this LOD are noted as <2 pg/mL.

#### C-reactive protein

CRP concentrations were measured using an automated turbidimetric assay (Roche Modular P800 module; Roche, Basel, Switzerland) with a lower detection limit (limit of detection, LOD) of 1 mg/L, which is routinely used in our laboratory.

### Statistical analysis

For statistical analysis, the Kolmogorov-Smirnov test was used to test for normality. Furthermore, we used independent sample t-test to test for a difference in means, Mann-Whitney U test as a non-parametric test and Pearson's χ^2^ test for binomial distributions. Significance was defined as p<0.05. Statistical analyses were performed using SPSS software for Windows (version 19; Lead Technologies, Charlotte, North Carolina, USA).

## Results

### Participants

**Figure 1:**
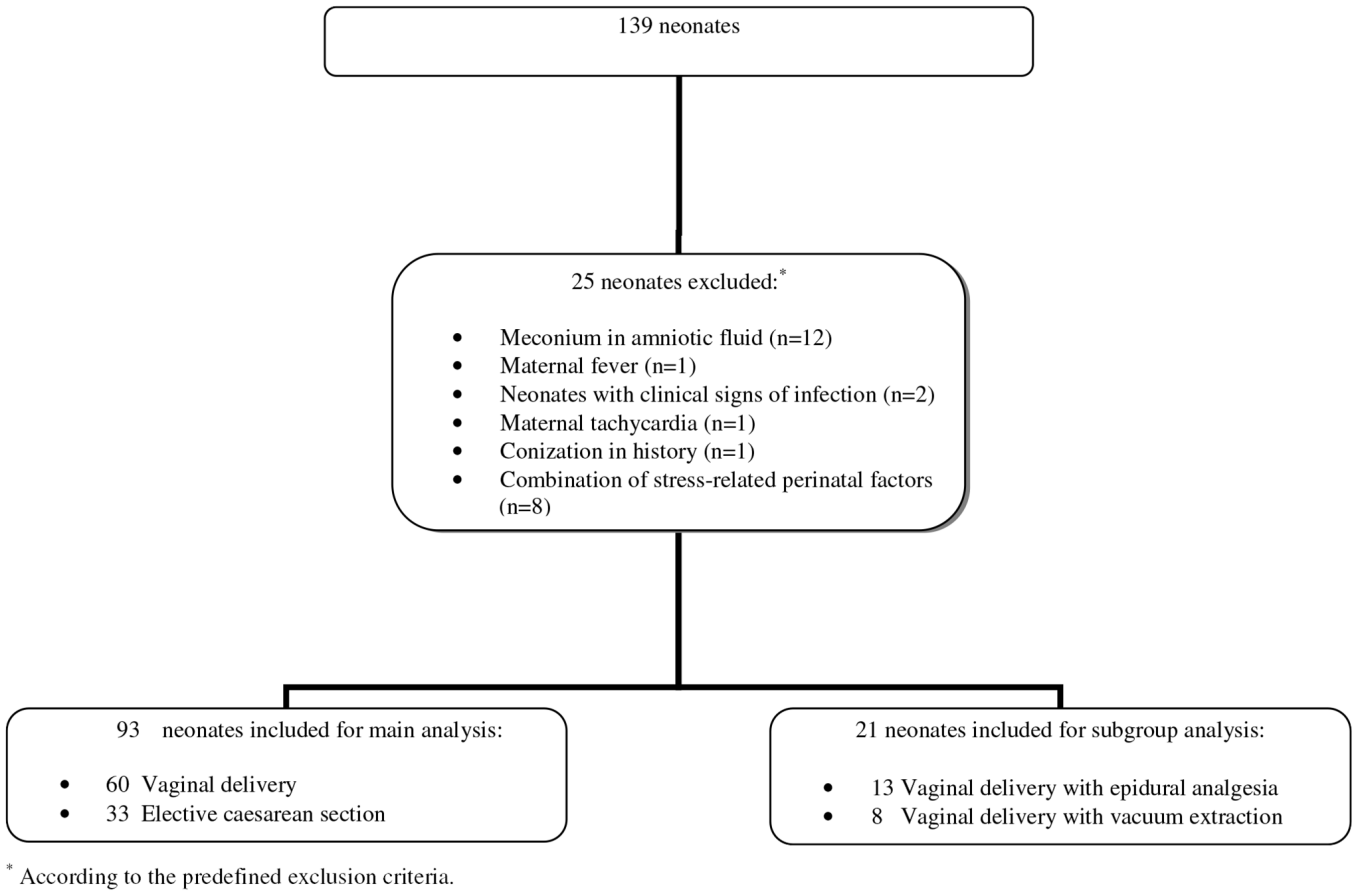
Flow diagram of enrolment.

Study enrolment and groups for the NESCIO Project are summarized in fig. 1. Cord blood samples were collected from 139 neonates: 25 of them were excluded, because they met any of the predefined exclusion criteria.

Table 1 shows the characteristics of 93 mother-child pairs belonging to two groups, i.e. vaginal delivery (n = 60) and ECS (n = 33). Some significant differences were observed between these groups. Gestational age was found to be shorter for infants delivered by ECS compared to vaginal delivery (39 vs. 40 weeks). Furthermore, children delivered by ECS were more likely to be female than those delivered by vaginal delivery (67 vs. 45%). In all neonates, CRP values in cord blood were below 1 mg/mL. The characteristics of the mother-child pairs of the subgroups vaginal delivery with epidural analgesia (n = 13) and vaginal delivery with vacuum extraction (n = 8) were comparable (data not shown). None of the children had developed fever 3 days postpartum.

**Table 1:** Characteristics of 93 mother-child pairs participating in the NESCIO Project.

	Vaginal delivery (n = 60)	Elective caesarean section (n = 33)	p Value
**Maternal characteristics**			
Age, years	31 (5)	32 (5)	0.443^1^
Gravida	2 (1)	2 (1)	0.930^2^
Para	1 (1)	1 (1)	0.586^2^
Gestational age, weeks	40 (1)	39 (1)	0.004^1^
Temperature before delivery, °C	36.6 (0.4)	36.7 (0.4)	0.255^1^
Time between sample collection and freezing, min	64 (21)	65 (18)	0.865^1^
*Medical indication, n (%)*			
Yes	51 (85)	33 (100)	
No	9 (15)	0	
*Pain relief, n (%)*			
Pethidin	21 (35)	0 (0)	
Regional anesthesia during elective caesarean section	0 (0)	33(100)	
None	39 (65)	0 (0)	
**Child characteristics**			
Birth weight, kg	3.603 (0.5)	3.521 (0.6)	0.485^1^
*Apgar score*			
1 min	9 (6–10)	9 (8–10)	0.364^2^
5 min	10 (8–10)	10 (9–10)	0.204^2^
10 min	10 (9–10)	10 (10–10)	0.132^2^
Temperature <1 h postpartum, °C	36.7 (0.4)	36.7 (0.4)	0.624^1^
*Sex, n (%)*			
Male	33 (55)	11 (33)	0.045^3^
Female	27 (45)	22 (67)	
*Admission to neonatology, n (%)*			
Yes	4 (7)	4 (12)	
No	56 (93)	29 (88)	

Values are given as mean (SD) unless stated otherwise.

1 Independent t-test,

2 Mann Whitney U Test,

3 Pearson's χ^2^ test.

**Figure 2:**
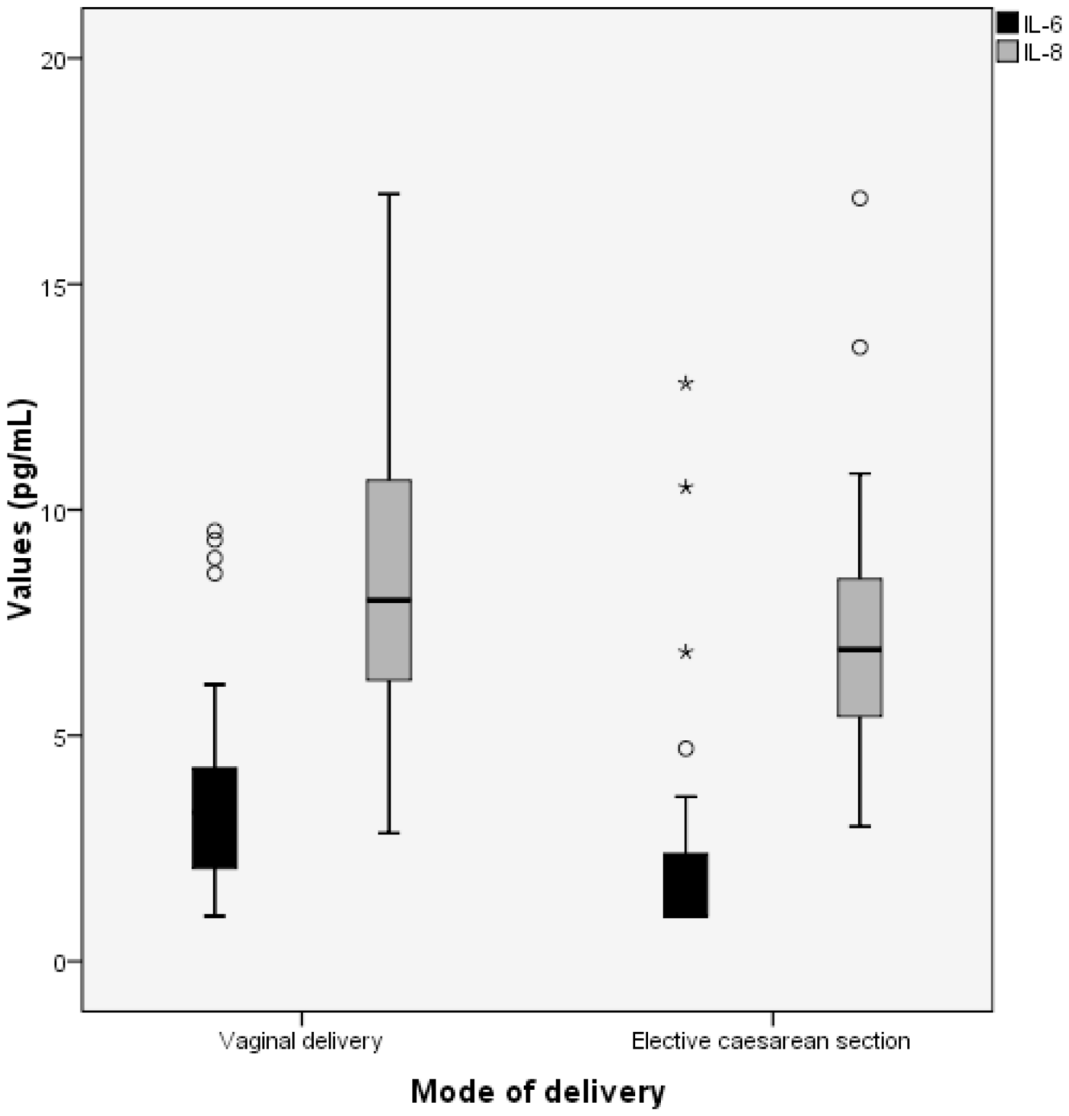
Comparison of interleukin-6 (IL-6) and interleukin-8 (IL-8) values in cord blood of healthy, term neonates delivered vaginally and by elective caesarean section.

IL-6 and IL-8 values in cord blood of the healthy term neonates are shown in Table 2 and fig. 2. In neonates delivered vaginally or by ECS, 23 and 63% of the IL-6 concentrations, respectively, were below the LOD (2 pg/mL) of the assay. These values occurred significantly more often in the ECS group (data not shown). For IL-6, an analytical method with a higher sensitivity than the Immulite 1000 used in this study is apparently not required as IL-6 values in EONS have been shown to be above 300 pg/mL (13,14). For statistical analysis in SPSS, the values below the detection limit were replaced by 1 (0.5xLOD), which did not influence the outcome. IL-6 values were not normally distributed according to the Kolmogorov-Smirnov test. Thus, median instead of mean values are provided for IL-6. In one neonate of the ECS group not showing any differences in prenatal or postnatal course compared with the other children a value of 48 pg/mL was measured. This value was considered to be an extreme outlier and omitted from further analysis. None of the IL-8 values in cord blood were below the LOD. IL-8 values were found to be normally distributed. Median values of IL-8 are only given to allow comparison with IL-6 levels in cord blood.

**Table 2:** Interleukin-6 (IL-6) and interleukin-8 (IL-8) values in cord blood of healthy, term neonates delivered vaginally and by elective caesarean section (ECS).

	Total (n = 93)	Vaginal delivery (n = 60)	ECS (n = 33)	p Value
**IL-6 (pg/mL)**				
Median (range)	2.4 (<2–12.8)	3.3 (<2–9.5)	<2 (<2–12.8)	0.001^1^
2.5^th^ percentile	<2	<2	<2	
97.5^th^ percentile	10.2	9.4	nd^3^	
**IL-8 (pg/mL)**				
Mean (range)	8.1 (2.8–17.0)	8.4 (2.8–17.0)	7.4 (3.0–16.9)	0.104^2^
Standard deviation	3.0	3.1	2.9	
Median	7.8	8.0 (2.8–17.0)	6.9 (3.0–16.9)	
2.5^th^ percentile	3.1	3.3	3.0	
97.5^th^ percentile	16.1	15.6	nd^3^	

1 Mann Whitney U Test,

2 Independent t-test,

3 nd  =  not determinable.

### Vaginal delivery

For vaginal delivery, the median concentration of IL-6 is 3.3 pg/mL with a range of <2 to 9.5 pg/mL. The mean value for IL-8 is 8.4 pg/mL with a range of 2.8 to 17.0 pg/mL.

### ECS vs. vaginal delivery

For ECS, the median concentration of IL-6 is <2 pg/mL with a range of <2 to 12.8 pg/mL. The median concentrations of IL-6 appeared to be significantly different between vaginal delivery and ECS (p = 0.001). The mean value for IL-8 is 7.4 pg/mL with a range of 3.0 to 16.9 pg/mL for ECS and showed no significant difference between vaginal delivery and ECS. For establishing reference values, these groups can be added together (n = 93). The mean value calculated was 8.1 pg/mL (SD 3.0 pg/mL).

### Subgroup analysis

Subgroups of newborns delivered vaginally combined with epidural analgesia or vacuum extraction were also included in this study. Although these groups were small (epidural analgesia, n = 13 and vacuum extraction, n = 8), a preliminary attempt was made to analyze the interleukin levels of these groups. Due to the small numbers, only median values are given for IL-6 and IL-8.

In neonates delivered vaginally combined with epidural analgesia, the median concentration for IL-6 is 4.0 pg/mL with a range of <2 to 9.1 pg/mL. This median value is not significantly different from the median value found in the group of newborns born via vaginal delivery without epidural analgesia (median: 3.3 pg/mL, range: <2–9.5 pg/mL). For IL-8, the median concentration is 8.7 pg/mL with a range of 3.1 to 13.6 pg/mL, which is also not significantly different from the reference group of vaginally delivered newborns (mean: 8.4 pg/mL, range 2.8–17 pg/mL). In neonates delivered vaginally combined with vacuum extraction, the median concentration for IL-6 is 5.3 pg/mL with a range of 2.2 to 13.7 pg/mL. This value showed a significant difference compared to the vaginal delivery reference group (p = 0.014). However, the median concentration for IL-8 is 8.4 pg/mL with a range of 4.7 to 16.0 pg/mL, which is not significantly different from the reference group.

## Discussion

In this study, we determined IL-6 and IL-8 reference values in cord blood of healthy term neonates and the influence of vaginal delivery vs. caesarean section and perinatal stress factors thereon. IL-6 is a cytokine of the early host response to infection, preceding the increase of CRP. IL-8 also belongs to the class of proinflammatory chemokines and has similar time kinetics as IL-6. The cytokines were measured by an automated solid-phase chemiluminescent immunometric assay, which is clinically useful because of the small volume required and quick reaction times.

In our study group, none of the neonates developed clinical signs of infection within 72 h postpartum. Gestational age was shorter for children delivered by ECS compared to vaginal delivery, probably caused by the fact that ECS was mostly scheduled at 39 weeks and spontaneous onset of labour occurred between 37 and 42 weeks. Furthermore, more female neonates were delivered by ECS, which was considered to be mere coincidence.

A mean value for IL-8 of 8.1 pg/mL with a standard deviation of ±3.0 pg/mL was found. Mode of delivery, i.e. vaginal delivery or caesarean section, had no significant influence on this value. Therefore, one reference value for IL-8 appears to apply to both groups. We propose a reference value of <14.1 pg/mL (mean + 2SD) for IL-8 in cord blood of healthy term neonates. For IL-6, we observed a significant difference between vaginal delivery and ECS. Since the actual difference is small compared to the IL-6 levels that are reported for neonates suffering from a neonatal infection (>300 pg/mL) [14], [16] we propose one reference value, being <10.2 pg/mL (97.5th percentile total group) for healthy term neonates born by vaginal delivery and ECS. The effects of maternal and perinatal variables on these proposed values merit further investigation.

We also measured interleukin levels in subgroups of newborns delivered vaginally combined with epidural analgesia or vacuum extraction to study potential influences of these perinatal stress factors on interleukin levels. Epidural analgesia per se does not appear to significantly influence cord blood IL-6 and IL-8 levels, while vacuum extraction is associated with a small increase in IL-6 levels. However, further research is needed to establish reference values for these groups, particularly for vaginal delivery with epidural analgesia. More and more women deliver under epidural analgesia. Although epidural analgesia is the superior mode of pain relief during labour a marked disadvantage is the fact that many women develop fever during labour. Because it is often difficult to distinguish between fever as side effect of epidural analgesia or fever as the first sign of intra-uterine infection with the possibility of neonatal infection and EONS, women with fever are often started on antibiotics during labour. Born neonates are admitted to the neonatal ward for continuation of antibiotic therapy until cultures prove to be negative. Median values of IL-6 and IL-8 for vaginal delivery with epidural analgesia did not show any statistical difference with median values for IL-6 and IL-8 for vaginal delivery without any perinatal stress factor. Therefore, an infectious origin of the fever that children often develop after vaginal delivery with epidural analgesia is unlikely. Determining IL-6 and IL-8 levels in cord blood may prove to be very useful in distinguishing between fever as side effect of epidural analgesia or fever as sign of EONS. This will be the subject of future studies.

The LOD of the analytical method for IL-6 and IL-8 in our study was 2 pg/mL. IL-6 values above the LOD occurred significantly more often in the vaginal delivery group compared to ECS. Contracting skeletal muscles can markedly enhance IL-6 levels [19], [20]. Therefore, the higher values of IL-6 found in the vaginal delivery group might well be due to uterine and skeletal muscle contractions, as previously suggested [21].

In the literature, several values have been reported for IL-6 and IL-8 in cord blood of term neonates determined by automated assays. Krueger et al. [16] found median IL-6 and IL-8 values of 13 and 21 pg/mL, respectively. In another study of 10 healthy term neonates, median IL-6 and IL-8 values of 4.75 and 20.0 pg/mL, respectively, have been reported [14]). These studies made no distinction between mode of delivery and the groups examined existed of premature neonates and term neonates. In general, all these values are in accordance with or in the same range as those found in our study. The results of some other studies on IL-6 and IL-8 values in preterm and term neonates are not comparable, as values were determined by manual assays and not by Immulite [3], [15], [17].

Based on the results of this study, we propose a reference value for IL-8 of <14.1 pg/mL in cord blood of healthy term neonates. One reference value for IL-8 applies to vaginal delivery as well as ECS. Regarding IL-6, we propose a reference value of <10.2 pg/mL. These values have to be validated in larger cohorts of neonates, inclusive of those with and without early-onset neonatal sepsis.
